# Preclinical Evaluation of a Potential GSH Ester Based PET/SPECT Imaging Probe DT(GSHMe)_2_ to Detect Gamma Glutamyl Transferase Over Expressing Tumors

**DOI:** 10.1371/journal.pone.0134281

**Published:** 2015-07-29

**Authors:** Harleen Khurana, Virendra Kumar Meena, Surbhi Prakash, Krishna Chuttani, Nidhi Chadha, Ambika Jaswal, Devinder Kumar Dhawan, Anil Kumar Mishra, Puja Panwar Hazari

**Affiliations:** 1 Division of Cyclotron and Radiopharmaceutical Sciences, Institute of Nuclear Medicine and Allied Sciences, Delhi, India; 2 Department of Biophysics, Biomedical Sciences Block, Panjab University, Chandigarh, India; Indian Institute of Toxicology Reserach, INDIA

## Abstract

Gamma Glutamyl Transferase (GGT) is an important biomarker in malignant cancers. The redox processes ensuing from GGT-mediated metabolism of extracellular GSH are implicated in critical aspects of tumor cell biology. Reportedly, Glutathione monoethyl ester (GSHMe) is a substrate of GGT, which has been used for its rapid transport over glutathione. Exploring GGT to be an important target, a homobivalent peptide system, DT(GSHMe)_2_ was designed to target GGT-over expressing tumors for diagnostic purposes. DT(GSHMe)_2_ was synthesized, characterized and preclinically evaluated *in vitro* using toxicity, cell binding assays and time dependent experiments. Stable and defined radiochemistry with ^99m^Tc and ^68^Ga was optimized for high radiochemical yield. *In vivo* biodistribution studies were conducted for different time points along with scintigraphic studies of radiolabeled DT(GSHMe)_2_ on xenografted tumor models. For further validation, *in silico *docking studies were performed on GGT (hGGT1, P19440). Preclinical *in vitro *evaluations on cell lines suggested minimal toxicity of DT(GSHMe)_2_ at 100 μM concentration. Kinetic analysis revealed transport of ^99m^Tc-DT(GSHMe)_2_ occurs via a saturable high-affinity carrier with Michaelis constant (*K*m) of 2.25 μM and maximal transport rate velocity (*V*max) of 0.478 μM/min. Quantitative estimation of GGT expression from western blot experiments showed substantial expression with 41.6 ± 7.07 % IDV for tumor. Small animal micro PET (Positron Emission Tomography)/CT(Computed Tomography) coregistered images depicted significantly high uptake of DT(GSHMe)_2_ at the BMG-1 tumor site. ROI analysis showed high tumor to contra lateral muscle ratio of 9.33 in PET imaging studies. Avid accumulation of radiotracer was observed at tumor versus inflammation site at 2 h post i.v. injection in an Ehrlich Ascites tumor (EAT) mice model, showing evident specificity for tumor. We propose DT(GSHMe)_2_ to be an excellent candidate for prognostication and tumor imaging using PET/SPECT.

## Introduction

Radiolabeled receptor targeting peptides have emerged as a class of radiopharmaceuticals for cancer diagnosis and therapy. The design of these radiopharmaceuticals require meticulous ligand design and metal coordination chemistry [1–2]. A large number of peptides have been designed and synthesized that are specific for certain receptors or target enzymes localized on the cell surface. Their expression has been found to be invariably high in diseased condition. The advantage of using peptides is their low molecular weight, easy diffusion to the target site and rapid clearance from the body [3]. Radiolabeled peptide chelator systems are used commonly to non-invasively visualize tissue over expressing the receptor or enzyme using molecular imaging modalities with high target to non target ratio. Ideally the radiopharmaceuticals intended for use in diagnosis should work at very low concentration (10^−6^ to 10^−8^ M) without any adverse pharmacological effect. Diagnostic radiopharmaceutical provides a non-invasive method for assessing and estimating the diseased condition by studying its distribution pattern and kinetics inside the body [4–7].

The enzyme Gamma Glutamyl Transferase (GGT) (GGT1, E.C. 2.3.2.2) is the key enzyme that catalyzes the breakdown of Glutathione (GSH) at the first step, forming a reaction intermediate whereby the γ-glutamyl group is bound to enzyme. In humans, GGT1 (hGGT1, P19440) is a cell-surface enzyme that hydrolyzes extracellular γ-glutamyl compounds, including GSH (γ-Glu-Cys-Gly) and leukotriene C4. GGT expression is considered to be early marker of neoplastic transformation. The increased expression of GGT in actively proliferating pre-neoplastic foci in the liver has been reported. Though the mechanisms underlying the increased GGT expression induced by carcinogens remained however unidentified [8]. A comprehensive analysis of GGT expression in 451 human malignant neoplasms has been documented previously [9]. Also, from the previous studies it is evident that the expression of GGT in cancer cells may represent an important factor in the appearance of a more aggressive (malignant) and resistant phenotype. Elevated hGGT1 activity in the serum is a common diagnostic marker of several diseases, including liver cancer, alcoholic hepatitis, disrupted bile formation, and pancreatic cancer [10–17].

GSH is a naturally occurring peptide and a substrate for GGT. GSH is stable in the intracellular milieu because of a unique amide bond between γ-carboxyl group of glutamate and amine group of cysteine [18, 19]. Many ester modifications of GSH have been synthesized [20]. Glutathione monoester (GSHMe) in which glycine carboxyl group has been esterified is previously demonstrated to have better permeability and transport into the cells than reduced GSH. It is found that there is more rapid transport of monoethyl ester of glutathione and that is ascribed to its higher hydrophobicity and increased bioavailability compared to glutathione [20–23]. In the present study, we have developed a peptide-chelator system, wherein the tripeptide is GSHMe which is conjugated to polyamino polycarboxylate chelator.

The bivalent approach was envisaged for designing the ligand based on tripeptide GSHMe for enhancing the binding affinity to GGT as compared to the monomeric glutathione. The expression of GGT is found to be high on various types of tumors viz. hepatocellular carcinoma, carcinomas of the skin, adenocarcinoma of the lungs, prostate carcinomas and in some mammary tumors types and is considered to be an early marker of neoplastic transformation. DTPA conjugated GSHMe, DT(GSHMe)_2_, is possibly transported via GGT system and could be an excellent candidate in imaging tumor and malignant lesions. In preclinical models, DT(GSHMe)_2_ has shown high and specific accumulation at tumor site versus inflammation. Even with a pharmacologically ineffective concentration of the tracer and lower dose of radioactivity, a good diagnostic image could be obtained using PET and SPECT. Therefore, it can be a better system for accurate interpretation of PET/SPECT (Single Photon Emission Computed Tomography) scans for differential diagnosis of GGT over expressing tumors. DT(GSHMe)_2_ could be promising candidate with prognostic value for translational molecular imaging of GGT over expressing tumors, plausibly with high utility in clinics with an easy kit based radiopharmacy.

## Materials and Methods

### Chemicals

Propidium iodide, Dulbecco’s Modified Eagle’s Medium (DMEM), RNAse, secondary Goat anti-Rabbit antibody, ECL reagent and Diaminobenzidine (DAB) were procured from Invitrogen, Carlsbad, CA, USA. Glutathione, Sulphuric acid, Diethylenetriaminpentaacetic acid (DTPA) anhydride, stannous chloride, sodium bicarbonate, 3, (4, 5-dimethylthiazol-2-yl)2, 5-diphenyltetrazolium bromide (MTT), Sulforhodamine B (SRB), Glacial acetic acid, Trichloroacetic acid (TCA), pyridine, Harri’s Hematoxylin, acetone, Xylene, DPX, sodium citrate, Trypsin-EDTA, Bovine serum albumin (BSA), Hank’s balanced salt solution (HBSS), Total Glutathione estimation kit were purchased from Sigma Aldrich, St Louis, MO, USA. Primary anti GGT polyclonal rabbit antibody was purchased from Abcam. Ethanol, methanol, dimethyl sulfoxide, dimethyl formamide, diethyl ether, triethylamaine were obtained from Merck specialities pvt. limited. All solvents used were of analytical grades. Radiocomplexation and the radiochemical purity were checked by instant thin layer chromatography (ITLC-SG) Strips from Paul Gelman, USA.

^99m^Tc was procured from Regional Center for Radiopharmaceuticals (Northern Region), Board of Radiation and Isotope Technology (BRIT), Department of Atomic Energy, India. ^68^Ga was eluted from in house ^68^Ge/^68^ Ga generator system (IGG100, Eckert and Ziegler, Berlin).

### Instrumentation

Mass spectra (ESI-MS in positive and negative ion mode) were performed on in-house Agilent 6310 system ion trap. Sample was run in mobile phase as a mixture of solvents where (A) 70% HPLC grade Water and (B) 30% Methanol (containing 0.01% TFA) in isocratic mode. Sample (1 mg/mL) was prepared in HPLC grade water containing 0.1% Formic acid with injection volume of 0.5 μL. HPLC analyses were performed on Agilent 1200 LC coupled to a UV detector (λ = 214 and 254 nm). The C-18 RP Agilent columns (5 μm, 4.6 mm × 250 mm, 5 μm, and 9.4 mm × 250 mm) were run on gradient mobile phase with a flow rate of 1mL/min. Flow cytometric measurements were carried out using FACS calibur flow cytometer (Becton Dickinson and Co., Franklin Lakes, NJ, USA). Radiolabeling counting was carried out using a well type gamma counter (Capintec). MTT assay absorbance and SRB absorbance were taken on Biotek Synergy H4 hybrid multiplate reader. In vivo PET imaging was performed using microPET imaging as a non-invasive technology. The acquisitions were performed using GE-FLEX Triumph microPET/SPECT/CT scanner. The state of the art scanner consists of a microPET module (LabPET4) with 2x2x10 mm^3^ LYSO/LGSO scintillators in an 8- pixel, quad-APD detector module arrangement. The micro-CT part consist of a high resolution micro CT tube with a focal spot size switchable between 10 or 50 μm. SPECT imaging was performed on Seimens Symbian T2 SPECT/CT and reconstructed using Seimens Syngo MI workplace.

### Cell Culture

The cerebral glioma cell line (BMG-1; DNA index = 0.96; wild type p53) was obtained from Bangalore, India and was maintained in house at 37°C in a humidified CO_2_ incubator (5% CO_2_, 95% air) in low glucose−DMEM. Cells were supplemented with 10% fetal bovine serum and regularly subcultured twice a week to maintain log phase growth of cells [24]. The Ehrlich Ascites tumor (EAT) cells (strain F-3) obtained from the Institute for Biophysics, University of Frankfurt, Germany, was maintained by serial passage of tumor cell suspension in the peritoneal cavity of BALB/c mice [25].

### Ethics Statement

All animal experiments and protocols were approved by the Committee on the Ethics of Animal Experiments of the Institute of Nuclear Medicine and Allied Sciences (INMAS), Defence Research and Development Organization (DRDO) (Institutional Ethical committee number under which this study has been approved is: 8/GO/a/99/CPCSEA). Animals were sacrificed using cervical dislocation method, when the tumor reached a volume not more than 10 cubic cm to avoid tumor burden related discomfort to the animal as per the UKCCCR guidelines for the welfare of animals in experimental neoplasia [26].

### Experimental Animals

Male BALB/c mice of age 6 to 8 weeks (average body weight 25 ± 2 gm) maintained on standard diet (Liptin, India) and water ad libitum were selected for experimental studies from an inbred colony in the animal house of the institute maintained at a constant temperature of 22 ± 2°C and 50% humidity under a 12 h light/dark cycles. Mice were housed in sterile plastic cages in specific pathogen-free conditions. Athymic mice were housed separately in isolator and fed well balanced sterile diet and water at 22 ± 2°C and 50% humidity under a 12 h light/dark cycles (with appropriate barriers, i.e., Air filters). Animals were anesthetized by inhalation of 2% isoflurane in 2 L/min oxygen.

### Xenograft models

Athymic male mice were xenografted with BMG-1 human tumor cell line (0.1 mL of cell suspension containing ~5 ×10^6^ BMG cells) in the flank region of the hind limb. BALB/c mice were inoculated with EAT murine tumor cells (5–6 × 10^6^) in the flank region of the right hind limb. An inflammation lesion was induced in the fore limb by intramuscular injection of turpentine oil (100 μL) 48 h before imaging. For Scintigraphy, (n = 6) mice used for imaging, biodistribution studies (n = 32 mice) for two experiments 4 mice/group for 4 time points and blood kinetics (n = 18).

### Synthesis and characterization

Glutathione monoethyl ester (GSHMe) was synthesized according to protocol as mentioned previously [27]. DT(GSHMe)_2_ has been synthesized using the methodology previously reported with slight modifications [28].

DTPA dianhydride (100 mg, 1eq) and GSHMe (329.4 mg, 3.5 eq) were dissolved in dry DMF (10 ml) and triethylamine (226.32 mg, 8 eq) was added to the suspension. Reaction was allowed to proceed at 65°C for 24 h under Nitrogen environment. TLC was performed (8:2; Acetonitrile: water) to monitor the completion of reaction. On completion, solvent was evaporated in vacuo. The crude product was precipitated with cold diethylether and the white product was extracted by filtration. The product was characterized with Mass spectroscopy (ESI/MS) and analytical HPLC.

Analytical HPLC: The mobile phase was (A) methanol and (B) water containing 0.01%TFA following the gradient elution technique for separation as 0–10 min 100–70% A, 10–20 min 70–50% A, 20–30 min 50–50% A. The flow rate for analytical HPLC was 1 mL/min

### Cytotoxicity studies of DT(GSHMe)_2_

#### Sulfo rhodamine B (SRB) assay

SRB assay was performed to estimate the cytotoxicity of DT(GSHMe)_2_. SRB binds to total protein content of the cells and is independent of metabolic activity. Cells were seeded at the density of 6000 cells per well in 96 well microtiter plate. Cells were treated with varying concentration of DT(GSHMe)_2_ from 0.001 μM-1000 μM for different time intervals (24, 48 and 72 h). At each time point, 100 μL of ice cold 10% (wt/vol) trichloroacetic acid (TCA) was added to each well and were incubated for 1 h at 4°C followed by washing the plate four times under running tap water. Plates were then air dried and 100 μL of 0.057% SRB solution was added in each well for 30 min. Plates were quickly rinsed four times with 1% (v/v) acetic acid and air dried. To develop color, 200 μL of 10 mM Tris base solution (pH 10.5) was added to each well and kept on gyratory shaker for 5 min. The color intensity was measured fluorometrically at excitation 488 nm and emission at 585 nm. Surviving fraction was calculated and plotted for the concentration range 0.001 μM-1000 μM as a function of time.

#### MTT assay

To confirm the results from SRB assay, MTT assay which measures cell metabolic activity was performed to correlate the results obtained using SRB assay. Exponentially growing cells were plated in a 96 well microtitre plate at a cell density of 4000 cells per well 24 h before treatment. Cells were treated with varying concentrations of the drug for different time intervals (24, 48, and 72 h). At the end of treatment, both the treated cells and untreated control were incubated with MTT at final concentration of 0.1mg/mL for 3 h at 37°C and the medium was removed thereafter. Cells from each treatment were lysed and the formazan crystals were dissolved using 150 μL of DMSO for 30 min. Optical density of extracts at 570 nm was measured (reference filter: 630 nm). Surviving fraction was determined and was plotted against concentration as a function of time.

#### Cell cycle analysis

10^5^ cells per well were plated in a Petri dishes and kept in the incubator overnight at 37°C. Following day, cells were treated with different drug concentrations (0.01 μM –1000 μM). At specific time points, media was carefully removed and cells were harvested in micro centrifuge tubes. Cells were centrifuged at 1200 rpm for 6 min. Supernatant was removed and 700 μL of 80% cold ethanol was added drop wise. Pellet was uniformly dispersed and samples were stored at 4°C for at least 24 h. Samples were centrifuged to remove supernatant and RNAse at a final concentration of 0.5 mg/mL was added to the pellet and incubated at 37°C for 1 h. Finally Propidium Iodide (PI) solution at a final concentration of 10μg/mL was added and the samples were analyzed by flow cytometry.

### Radiolabeling of DT(GSHMe)_2_ with ^99m^Tc and ^68^Ga

#### ^99m^Technetium labelling

^99m^Tc radiolabeling was optimized for various concentrations of stannous and pH.

In an evacuated vial, 100 μL of DT(GSHMe)_2_ (100 nmoles) was taken and optimized 75 μg of freshly prepared SnCl_2_ (1 mg/mL of SnCl_2_ in 10% acetic acid) was added. The pH of the reaction mixture was adjusted to ~ 7.0 with 0.5 M Na_2_CO_3_. 500 μL of ^99m^Tc-pertechnate (~370±10 MBq) was added and incubated at room temperature for 30–40 min [28].

Radiochemical purity was determined using the percentage of free ^99m^Tc pertechnetate, hydrolyzed and reduced to that of the complex (conjugated with the ligand). Stability of the ^99m^Tc-DT(GSHMe)_2_ in saline was monitored using ascending thin layer chromatography using ITLC strips at different time intervals (1, 2, 4 and 24 h).

#### ^68^Gallium labelling

DT(GSHMe)_2_ was radiolabeled with ^68^Ga according to the protocol as previously reported with slight modifications [29–30]. The pH of ^68^GaCl_3_ eluate (~ 260 ± 7.8 MBq) from the generator, was adjusted to ~ 4.8 with sodium acetate and (100 nmoles) of DT(GSHMe)_2_ dissolved in deionized water was then added. The mixture was then heated to 75–80°C for about 10 min, allowed to cool and passed through an activated C-18 cartridge. After washing, purified tracer was eluted with 2 mL of ethanol. Solvents were eliminated by heating at 80°C for 5 min and ^68^Ga-DT(GSHMe)_2_ was reconstituted in 0.9% saline for injection. Radiochemical purity was assessed by Radio TLC and Radio HPLC.

### Log P calculation

The value of partition coefficient of radiolabeled DT(GSHMe)_2_ is calculated and expressed as log P. The radiolabeled DT(GSHMe)_2_ was passed through 0.22 μ filter and approximately 10.2 ± 1.5 MBq of labeled compound was diluted in PBS, pH 7.4. To this equal volume of 1-octanol was added. The mixture was thoroughly vortexed for 10 min and then centrifuged at 5000 rpm for 5 min. The counts in 150 μL, 100 μL and 50 μL samples of both organic and inorganic layers were determined by a well type gamma counter. The measurement was repeated thrice. The partition coefficient (P) was calculated using the following equation wherein, P = (counts in octanol fraction) / (counts in PBS fraction). For each value of P, log P value is calculated.

### Cell Binding Studies

The specificity of ^99m^Tc-DT(GSHMe)_2_ to bind externally on tumor cells were examined by receptor binding assays on EAT cells isolated from the ascitic fluid of BALB/c Mice. Cells were diluted to 2 x 10^6^ cells per mL and were aliquoted in different micro centrifuge tubes. Cells were washed twice with HBSS and finally resuspended in 1 mL of HBSS. Cells were then treated with various concentration of ^99m^Tc-DT(GSHMe)_2_ (10^−7^ M– 10^−12^ M) for 2 h at 37°C. Binding experiments were conducted in triplicates at 37°C both in the absence and presence of the 100-fold excess unlabeled glutathione to estimate the total binding and nonspecific binding respectively. Specific binding was obtained by subtracting nonspecific binding from total binding. At the end of each incubation time, the cells were washed with cold PBS twice and pelleted down. Finally, cells were lysed and cell-associated radioactivity was determined by gamma counting. The total protein content of cells was estimated by Bicinchoninic acid (BCA) method. Similarly the binding experiments were performed on monolayer of BMG-1 cells (2 x 10^6^) and were treated with ^99m^Tc-DT(GSHMe)_2_ (10^−7^ M– 10^−12^ M) for 2 h at 37°C. Binding experiments were conducted in triplicates at 37°C in the absence and presence of the 100-fold excess unlabeled glutathione to estimate the total binding and nonspecific binding respectively. Cells were lysed and cell associated radioactivity was determined. The total protein content was determined by BCA method. Scatchard Plot were plotted to determine Kd and Bmax.

### Time course and Kinetics of ^99m^Tc-DT(GSHMe)_2_

EAT cells were isolated from ascitic fluid of BALB/c mice and were diluted to 3 x 10^6^ cells/mL. Cells were aliquoted in different micro centrifuge tubes and were treated with 200 μM of ^99m^Tc-DT(GSHMe)_2_ for different time intervals (0, 10, 30 sec, 1, 2, 5, 10, 30 min, 1 and 2 h). After the treatment, cells were centrifuged at 2000 rpm for 5 min and supernatant was carefully removed. Followed by three washes of cold saline and acidic wash buffer, cells were centrifuged to yield the pellet. The pellet was dissolved in 500 μL of lysis buffer. The amount of radioactivity (CPM) in cell lysate was determined by gamma counter. Later, after the activity decayed, total protein from the cells was estimated by BCA method of protein estimation. Absorbance values were determined at 595 nm on a multi plate reader. A standard plot was constructed taking various dilutions of BSA. Drug uptake was calculated and plotted.

### Glutathione Estimation from Tumor, Blood and Plasma

Levels of total Glutathione (GSH/GSSG) were estimated from tumor, RBC and plasma isolated from BALB/c mice bearing EA tumors, after injection of unlabeled 100 μM DT(GSHMe)_2_ (100 μL). At time points (15 min, 30 min, 1, 2and 4 h), blood samples were withdrawn and tumors were excised. Tumors were homogenized in 3 volumes of 5% sulfosalicylic acid (SSA) and centrifuged at 10,000 x g for 10 min at 4°C. Blood was centrifuged and the plasma fraction was separated from RBC pellet. To each RBC and plasma fractions, equal volume of 5% SSA was added, followed by vortexing and centrifugation at 10,000 x g for 10 min at 4°C. Supernatant was collected and used for estimating total glutathione levels.

### Immuno-histochemical Analysis

The expression of GGT in the tumor was confirmed by performing immuno-histochemical staining for GGT on tumor tissue excised from xenograft tumor models. Briefly, the tissues were rinsed in PBS twice and fixed in 10% buffered formalin for 24 h. Paraffin embedding and sectioning (3 μ) was done. Sections were probed for the expression of GGT by incubating with primary rabbit anti-GGT1 polyclonal antibody (Abcam, Dilution 1:100) at 4°C overnight. Detection of Primary antibody was performed using HRP-conjugated goat anti-rabbit antibody (Santa Cruz, Dilution (1:500). Diaminobenzidine (DAB) was used as a chromogen and sections were counter stained with Harris’s hematoxylin.

### Western Blotting

Western blotting was done to confirm the expression of GGT on different tissue types (liver, tumor and contralateral muscle). All three tissues were excised from BMG-1 xenografted models and were equally weighed (5 mg). Tissues were homogenized in RIPA buffer containing protease inhibitors (aprotonin, PMSF, leupeptin) and centrifuged at 12000 rpm for 20 min. Supernatant was carefully aspirated and protein concentration was measured in each sample by the BCA protein estimation method. Briefly, 20 μg of denatured protein lysate was loaded on a 12% SDS-PAGE. The protein bands were blotted onto a nitrocellulose membrane and probed for GGT and GAPDH using specific primary antibodies. This was followed by treatment with the appropriate peroxidase-conjugated secondary antibody and visualized by ECL reagent (Invitrogen, Life Technologies). The chemiluminescence signal was captured using ChemiDoc (DNR Bio-systems) and Alpha View FC2 software (Alpha Innotech) was used to quantify the signal intensity of the bands. The band intensity of the target protein is expressed as % integrated density value.

### In silico Docking Studies of DT(GSHMe)_2_ on GGT

All the computational studies were performed using Schrödinger Software; Maestro 9.7 (Schrödinger, LLC, New York, NY, 2014); maestro 9.5 Prime version 2.3, Ligprep Version 2.9, Glide version 6. The crystal structure of hGGT; PDB: 4GG2 was subjected to Protein Preparation Wizard which includes pre-processing by assigning bond orders, addition of hydrogen and deletion of water molecules beyond 5 Å from hetero (het) groups and further minimization and optimization of protein structure was performed in the presence of force field OPLS2005. Induced fit docking studies were performed for DT(GSHMe)_2_ and Re-DT(GSHMe)_2_ by using coordinates of crystal structure of the human GGT1(hGGT1, PDB: 4GG2). The potential substrate binding site was identified and grid was prepared without any constraints. Docking analysis was processed after grid generation on hGGT, 4GG2 at three key regions within the active site: (a) the glutamate side chain including residues Arg-107, Ser-451, Ser-452,and Thr-399 (b) the side-chain of the catalytic nucleophile (Thr-381), and (c) the tripeptide (Gly-473, Gly-474, and Thr-475) that forms the oxyanion hole. Scaling factors were not applied to the Vander Waals radii and no constraints were applied. Default settings were used for all the remaining parameters for induced fit docking of DT(GSHMe)_2_ and Re- DT(GSHMe)_2_.

### *In vivo* studies on experimental models

#### Scintigraphy

Xenografted experimental models were intravenously administered 0.1mg/Kg body weight ^68^Ga-DT(GSHMe)_2_ (37 ± 2.2 MBq) and 0.25 mg/Kg Body weight ^99m^Tc-DT(GSHMe)_2_ (29.6 ± 1.8 MBq) via tail vein for PET and SPECT respectively. Blocking dose of ~ 5 mg/Kg body weight of GSH was administered intravenously in the experimental animal. The acquisition was performed 2 h post injection. The ratios were calculated from counts per pixel of individual ROI (Region of interest) for semi-quantitative analysis to understand the distribution and localization.

#### Biodistribution studies and blood kinetics

Radiolabeled ^99m^Tc-DT(GSHMe)_2_ (0.25 mg/Kg body weight) in a volume of 150 μL (11.1 ± 1.2 MBq) was carefully injected through the tail vein of each BALB/c mice xenografted with EAT Tumors in the hind limb. At 30 min, 1, 2 and 4 h post injection mice were sacrificed and different organs and tissues were dissected out. All the organs and tissues were rinsed in saline, weighed and counted in a gamma counter. Uptake of the radiotracer in each tissue was calculated and expressed as a percentage injected dose per gram of the tissue (% ID/g) and corrected for ^99m^Tc decay.

Biodistribution studies were also performed on tumor bearing BALB/c mice. 0.1 mg/Kg body weight ^68^Ga-DT(GSHMe)_2_ (~25 ± 1.5 MBq) was injected through tail vein and at different time points viz., 30 min, 1 and 2 h animals were sacrificed and organs were dissected out. Uptake of radioactivity in individual organ is noted and %ID/g is then calculated.

Blood clearance was performed to observe the rate of clearance of drug post administration. Radiolabeled conjugate 0.25 mg/Kg body weight of ^99m^Tc-DT(GSHMe)_2_ (22.2 ± 2.8 MBq) was administered intravenously through the tail vein of the mice. Blood samples were withdrawn at different time intervals (5, 15, 30 min, 1, 2 and 24 h). Decay corrected radioactivity is expressed as % ID, assuming a weight based theoretical blood volume as 7% of the body weight.

## Results

### Synthesis of GSH mono-ethyl ester and DT(GSHMe)_2_

The synthetic scheme involves mono-esterification of GSH in ethanol using a catalytic amount of sulfuric acid. Amount of sulfuric acid and temperature of the reaction was carefully adjusted so as to deter the formation of diethyl ester of glutathione. The yield of the product after the reaction completion was 87%. Mass spectroscopy of the compound confirmed the synthesis of the product. MS, *m*/*z* calculated for C_12_H_21_N_3_O_6_S: 335.12 was found at [M+H] 336.3. Mass spectrum is given in supplementary information

**Fig 1:**
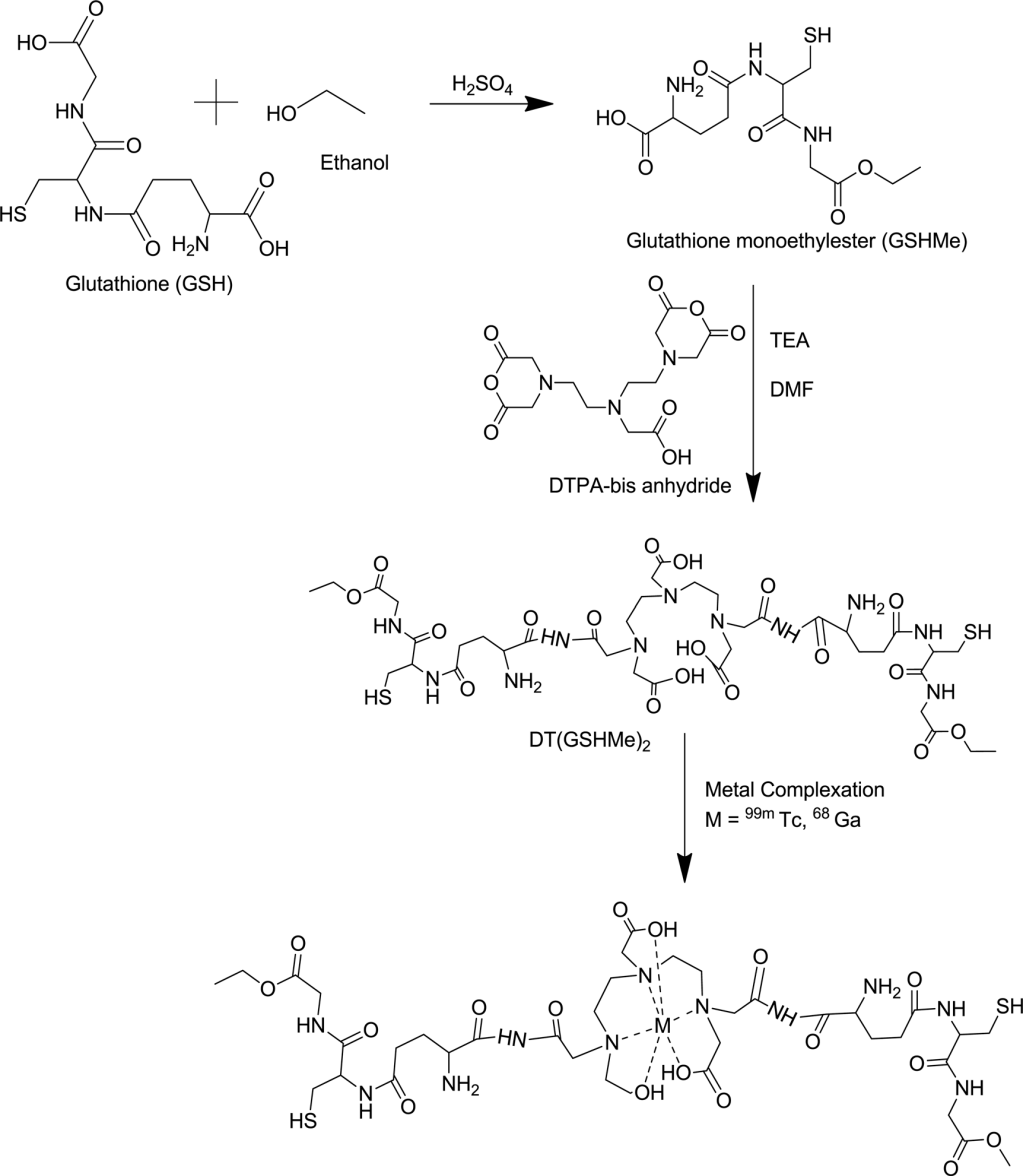
Schematic representation for synthesis of GSHMe and DT(GSHMe)_2_.

The procedure for the synthesis of DT(GSHMe)_2_ was accomplished by a well established methodology for the synthesis of DTPA conjugates. The procedure was followed with some modification of the molar ratios and base which considerably improved the yield of DT(GSHMe)_2_ conjugate. (Fig 1)

The yield of the Final product was 82.5% (n = 10). The final purified product was characterized using mass spectroscopy. MS, *m/z* of DT(GSHMe)_2_ calculated for C_38_H_61_N_9_O_20_S_2_: 1027.35 was found at [M+H] 1028.4 (Mass spectrum in supplementary information). Analytical HPLC was performed for DT(GSHMe)_2_ on a gradient of Methanol: Water. Peak of DT(GSHMe)_2_ was observed with retention time of 5.89 min with 66.16% purity (see supplementary information for chromatogram).

### *In vitro* cytotoxicity evaluation of DT(GSHMe)_2_

#### MTT assay

**Fig 2:**
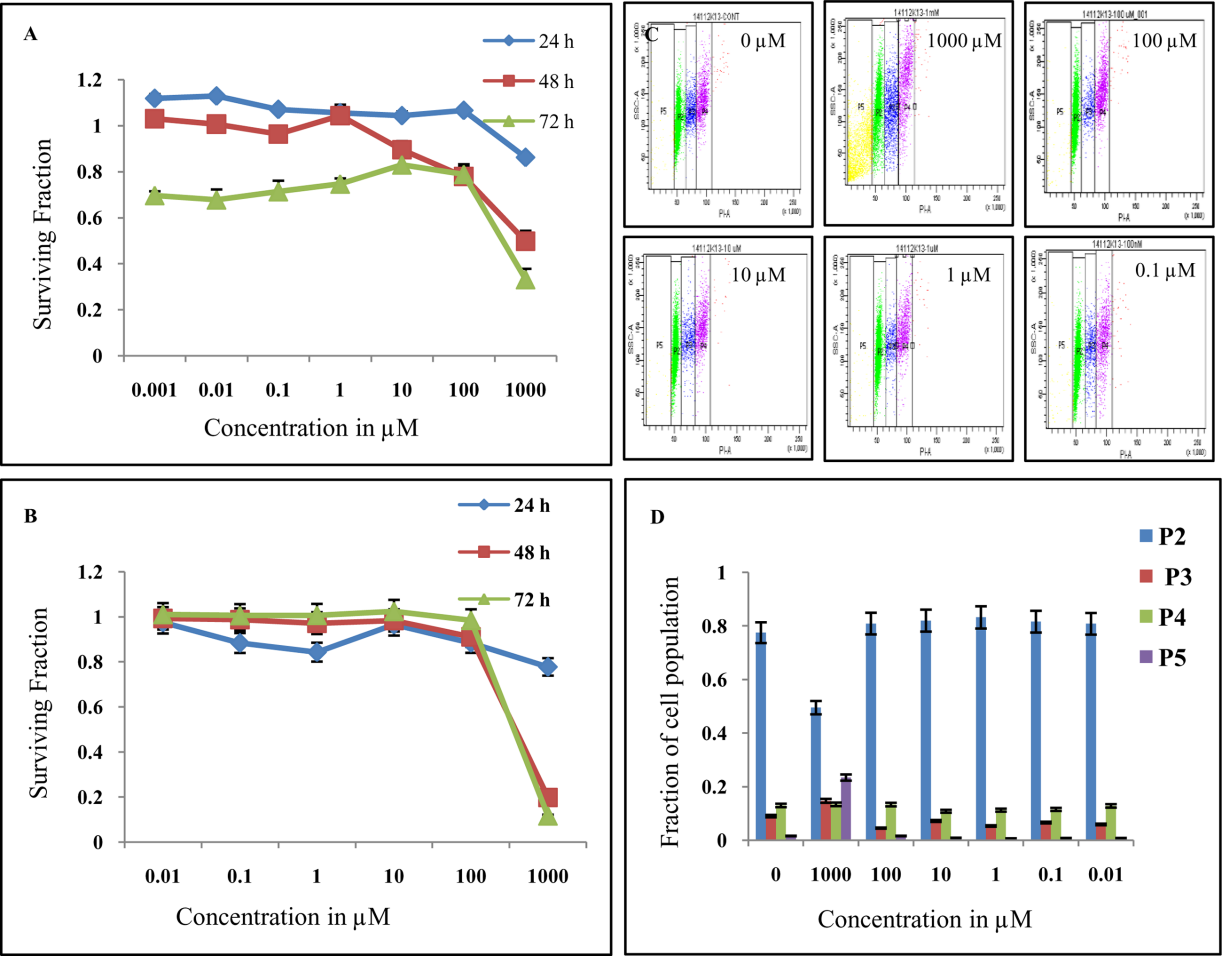
*In vitro* cytotoxic evaluation of DT(GSHMe)_2_ A&B) Data is plotted for surviving fraction versus concentration for different intervals. A) Represents MTT and B) represents SRB assay showing similar trend in the surviving fraction. C&D) Cells treated with different concentrations of DT(GSHMe)_2_ analyzed for cell cycle by flow cytometry. C) Gated population P2, P3, P4, P5 representing cells in G1/G0, S, G2/M and apoptotic cells respectively. B) Bar graph depicting fraction of cells in different phases (P2, P3, P4, P5) of cell cycle.

Cell viability was assessed by calculating surviving fraction for various drug concentrations (0.001 μM- 1000 μM) for different time intervals 24, 48 and 72 h. Fig 2A shows time dependent curve which depicts both concentration and time dependent toxicity. The drug was found to be nontoxic at lower concentration, but showed a fall in cell viability from concentration 100 μM and above. Significant cell death about 70 ± 4.38% was observed at 1000 μM at 72 h. IC_50_ of DT(GSHMe)_2_ was found to be approximately 500 μM.

#### SRB assay

Data is plotted for surviving fraction versus concentration for different time points as depicted in Fig 2B. In SRB assay also the drug was found to be nontoxic at lower concentration, but a sudden decline in surviving fraction was observed at 100 μM. Only 11.57 ± 3.84% of the cell population remained viable at 72 h time point for 1000 μM concentration. Nearly 50% cell lysis was observed at 500 μM concentration which gives its IC_50_ value.

Both MTT and SRB assays showed a similar trend in the surviving fraction of the cells after treatment which corroborates the correlation of both the assays.

#### Cell cycle analysis

Cell cycle analysis was performed on BMG-1 cells after 24 h treatment with DT(GSHMe)_2_ for various drug concentrations from 0.01 μM to 1000 μM. It was observed from Fig 2C and 2D, that there was a significant increase of apoptotic cells, approximately 14 folds, in 1000 μM concentration treatment i.e. 23.4 ± 1.17%, in comparison to control having 1.6 ± 0.08% cells undergoing apoptosis. A substantial decrease in G1 cell population was observed, in cells treated with 1000 μM drug concentration with remaining 49.5 ± 2.47% cells as compared to control with a value of 77.5 ± 3.87%. A slight increase in G1 cell population, 83.2 ± 4.16% was seen in cells treated with 1 μM DT(GSHMe)_2_ when compared with control 77.5 ± 3.87%.

### Radiochemistry of DT(GSHMe)_2_ and Quality analysis

#### ^99m^Tc-DT(GSHMe)_2_

Reducing agent SnCl_2_ was used to convert NaTcO_4_^-^ to electro positive cationic ^99m^Tc form which then form complex with DT(GSHMe)_2_. The quality control of the radiocomplexation of ^99m^Tc labeled DT(GSHMe)_2_ was done by instant thin layer chromatography using different solvent systems i.e. Acetone, PAW (Pyridine: Acetic acid: Water in the ratio 3:5:1.5) and Saline. The conjugate shows >98% complexation with ^99m^Tc with negligible free ^99m^Tc in the solvent front (< 2% without purification). The presence of technetium colloids is estimated by running the TLC in PAW as the solvent system. As little as 0.4 ± 0.25% colloids were estimated which remain bound at the origin and were found as traces when ^99m^Tc formed complex with DT(GSHMe)_2_. Negligible amounts of hydrolyzed technetium were found at the origin when TLC was run in saline.

#### ^68^Ga-DT(GSHMe)_2_

The overall radiochemical yield of ^68^Ga-DT(GSHMe)_2_ was ~65% (n = 8). To confirm the identity of radiochemical product, Radio-HPLC was performed with methanol: water (60:40) as solvent. Concurrent peak in the HPLC UV detector and the HPLC gamma detector at Retention time 3.5 min confirmed the labeling with ^68^Ga. (See supplementary information for Radio HPLC and TLC.)

### Determination of Log P

The results demonstrated Log P of -1.313 ± 0.104 for ^99m^Tc-DT(GSHMe)_2_ whereas Log P value of glutathione of -3.23 ± 0.141 thereby decreased in the hydrophilicity of the conjugate could be attributed to its ester modification.

### Cell Binding Studies of ^99m^Tc-DT(GSHMe)_2_

**Fig 3:**
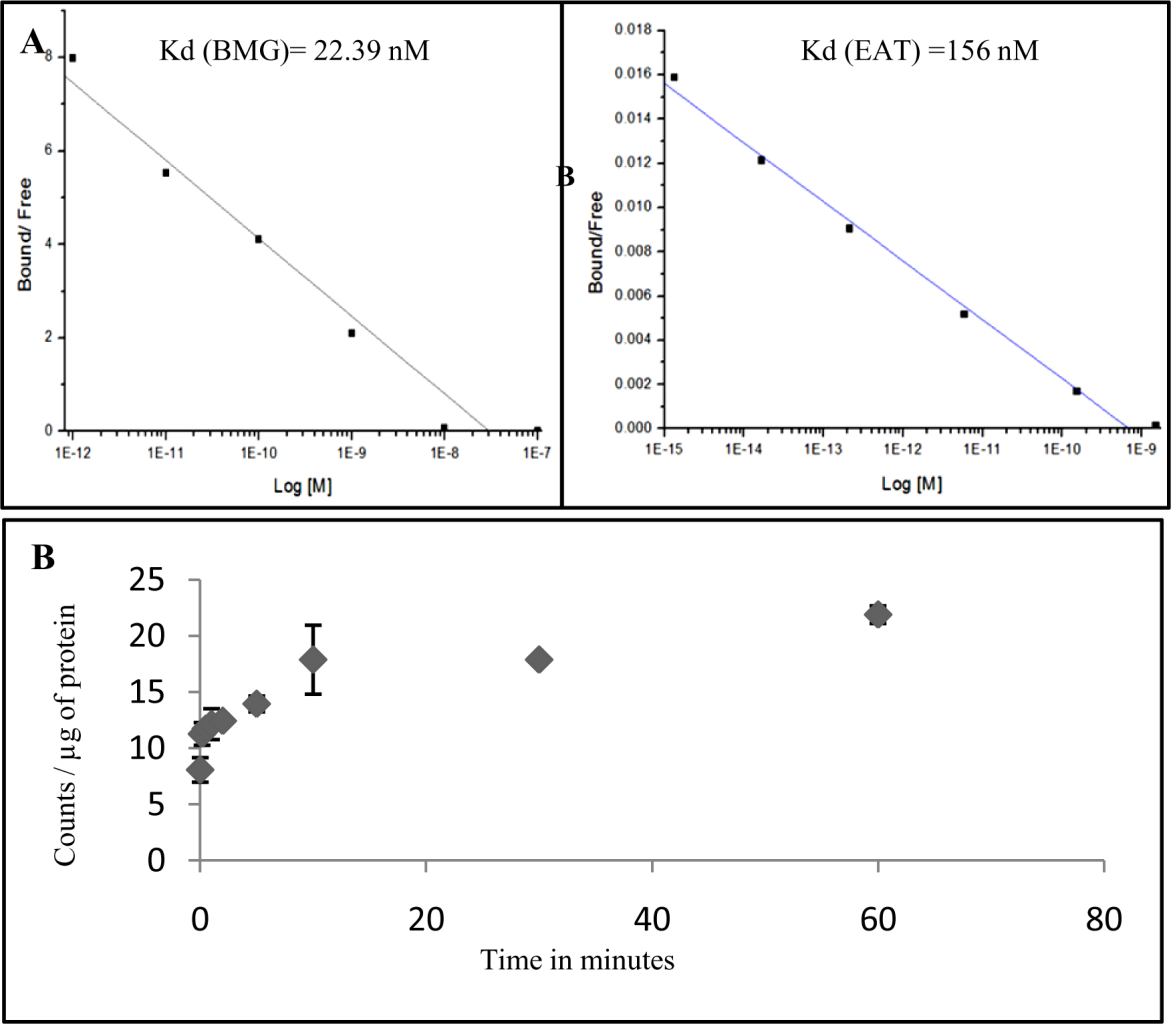
Cell binding assay and uptake kinetics of DT(GSHMe)_2_ A&B) Representative scatchard plot resulting from competitive binding assay of ^99m^Tc-DT(GSHMe)_2_ on BMG-1 and EAT cells. Resulting Kd of 22.9 nM and 156 nM was observed in BMG-1 and EAT cells respectively C) Graphical representation of time course and kinetics of ^99m^Tc- DT(GSHMe)_2_ on EAT cells.

The ability of ^99m^Tc-DT(GSHMe)_2_ conjugate to bind target enzyme GGT on the surface of tumor cell lines was examined by binding assay. Nonspecific binding was determined using 100-fold excess of unlabeled glutathione. Examination of binding curves showed significant external binding of the labeled ^99m^Tc-DT(GSHMe)_2_ conjugate. Fig 3A and 3B shows the scatchard plot of DT(GSHMe)_2_ on BMG and EAT cells respectively. The Scatchard plot shows affinity of the labeled ^99m^Tc-DT(GSHMe)_2_ on tumor cells. Kd was derived graphically and was found to be 156 nM in EAT tumor cells and 22.39 nM in BMG cells respectively. Bmax was found to be 17.2 nM and 91.02 nM for BMG-1 and EAT cells respectively.

### Time Course and Kinetics of ^99m^Tc-DT(GSHMe)_2_

Time course experiments revealed that transport of ^99m^Tc-DT(GSHMe)_2_ into cells was rapid and linear for up to 30 min, saturating thereafter, and reached a plateau after 2 h of incubation as seen in Fig 3C. Kinetic analysis revealed that transport of ^99m^Tc-DT(GSHMe)_2_ occurs via a saturable high-affinity carrier with Michaelis constant (*K*m) of 2.25 μM and maximal transport rate velocity (*V*max) of 0.478 μM/min. A single linear regression line was obtained in the Eadie-Hofstee transformation indicative of the function of a saturable system or systems with comparable functional characteristics.

### *In Vivo* Total Glutathione Estimation

Levels of total Glutathione were estimated from the tumor tissue excised from the xenografted animal models and was expressed as μmoles/ g tissue weight for different time intervals post intravenous injection of 100 μM of unlabeled DT(GSHMe)_2_.

Further, levels of total glutathione were also checked in RBC and plasma fractions of blood and were expressed as μmoles/mL of the sample. A distinctive trend in the levels of total GSH is observed in tumor, RBC and Plasma. It was observed that the levels of total GSH in tumor tissue gradually increased from 3 ± 0.007 μmol/g tissue weight at 0.25 h and maximizes up to 4.9 ± 0.010 μmol/g tissue weight at 2 h followed by a decline in the levels by 4 h. DT(GSHMe)_2_ provides free thiol groups which react with DTNB to give a yellow coloured product (TNB) in the presence of NADPH, which is measured spectrophotometrically The trend corroborates the result of biodistribution studies which depict the organ bound activity with respect to time. (Table 1)

**Table 1:** Quantifying the intracellular Glutathione in different tissues as a function of time.

Time (h)	Tumor (μmoles of GSH/g tissue weight)	RBC (μmoles of GSH/ml)	Plasma (μmoles of GSH/ml)
0.25	3 ± 0.007	0.38 ± 0.021	0.13 ± 0.029
0.5	4.3 ± 0.019	0.39 ± 0.026	0.16 ± 0.028
1	4.5 ± 0.070	0.45 ± 0.010	0.28 ± 0.127
2	4.9 ± 0.010	0.5 ± 0.11	0.32 ± 0.155
4	4.1 ± 0.010	0.39 ± 0.001	0.23 ± 0.041

Depicting the levels of total glutathione derived from tumor, RBC as a function of time and plasma after injection of 100 μM of unlabeled DT(GSHMe)_2_; n = 3

### Studying the expression levels of GGT in tumors

**Fig 4:**
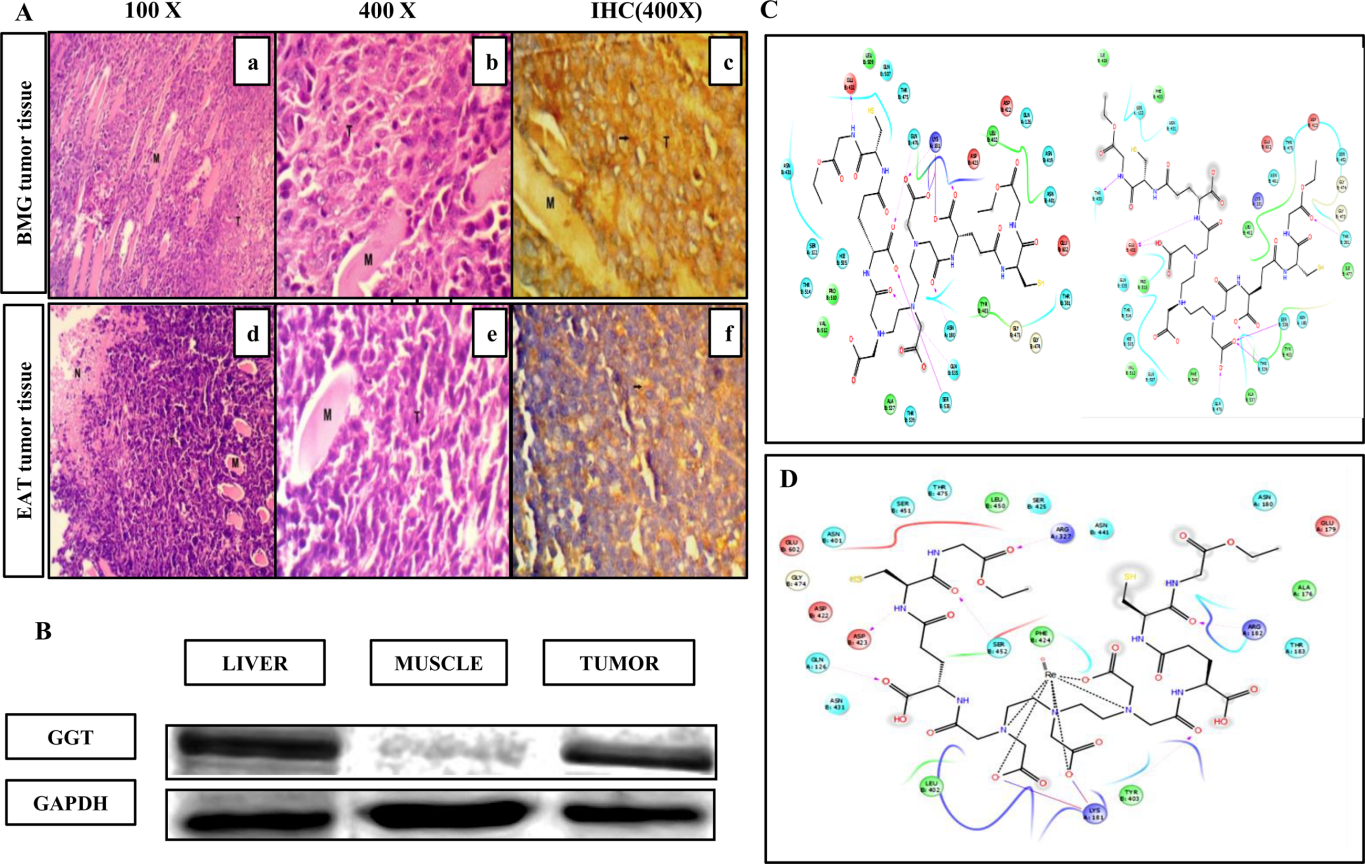
Estimating the expression of GGT ex vivo and in silico docking studies A)The upper panel images shows BMG-1 tumor tissue histology (a & b) 100X and 400 X magnification micrograph of H& E stained tissue sections. (c) IHC to probe GGT expression on BMG-1 tumor tissue, 400 X. Lower Panel images show EA tumor histological images. (d & e) 100X and 400X magnification images of H & E stained EA tumor tissue sections (f) 400X image showing moderately positive expression of GGT in EA tumor. B) Representative western blot showing the expression of GGT and GAPDH in three tissue types (Liver, muscle, tumor). C) Two dimensional view of two conformations of DT(GSHMe)_2_ and D) Re-DT(GSHMe)_2_ with 4GG2 where green color represent hydrophobic, light blue are polar, red as positive charged residues.

To evaluate the expression of GGT on tumor tissues derived from BMG-1 and EAT cells immuno-histochemistry with anti-GGT polyclonal antibody was carried out and analyzed. GGT expression was dramatically increased in BMG-1 tumor tissue as seen in Fig 4A (c). High magnification micrograph from the edge of the BMG-1 tumor implant showed tumor cells infiltrating into host muscle tissues as seen in Fig 4A (a). The tumor cells having large vesicular nuclei with pleomorphic nuclear forms were observed. Whereas in EAT tumors, Fig 4A (f) showed moderate positivity for GGT in the cytoplasm of the tumor cells. Also Section from EAT tumor depicted necrosis (N) on the left edge. Since high expression of GGT was observed on excised BMG-1 tumors in IHC, further validation was done by western blotting. As depicted in the Fig 4B, the expression of GGT is visibly observed in liver and tumor tissue. Liver tissue expresses GGT and is taken as positive control. The levels of GGT were significantly higher in tumor as compared to muscle which expresses at basal level. Densitometric analyses were done using Alpha View FC2 software (Alpha Innotech). The band intensity of the target protein is expressed as % integrated density value (% IDV) which is 41.6 ± 7.07% for tumor, 15.9 ± 6.64% for muscle and 52.75 ± 0.775% for liver tissue. Approximately 2.6 fold higher expression of GGT in tumor as compared to that of muscle tissue was observed.

### Theoretical evidence of Binding of DT(GSHMe)_2_ on GGT

The characterization of crystal structure of hGGT has opened the avenues for studying the binding interactions of molecules designed for enhanced diagnostic and therapeutic efficacy of different cancer types. Docking studies were performed for DT(GSHMe)_2_ and Re-DT(GSHMe)_2_ with GGT, 4GG2 at active site. Re and Tc complexes show same size, geometry, dipole moment, formal charge, ionic mobility, etc. and can be safely anticipated to be analogous [31].

The two dimensional view of interactions diagram (Fig 4C) shows the good binding of DT(GSHMe)_2_ at the active binding site in two different conformations with one of the GSHMe moiety binding at the substrate binding loop. The high G score of -7.518 signifies dominant hydrogen bond interactions to side chains and backbone of residues (Gln 476, Gln 535, Ser 538, Lys 181 and Asn 180) with carboxylate and carbonyl groups in the ligand and –NH was found to be hydrogen bonded to Glu 432 side chain.

Another conformation with G score -4.414 shows that ligand resides within the vicinity of active site residues. This catalytic nucleophilic residue Thr 381 interacts with the carbonyl oxygen of GSHME, which is a significant interaction for DT(GSHMe)_2_ to be cleaved at the active site. In similar terms, Re-DT(GSHMe)_2_ (G score -5.38, Fig 4D) was found to be at the substrate binding pocket with conserved residues. The docking pose in both the cases results in occupancy of ligand and metal complex at the substrate binding site. The docking results shed light into the potential of DT(GSHMe)_2_ and Re-DT(GSHMe)_2_ being the substrate for GGT and thus effective for targeting tumor cells [32–35].

### Biodistribution and Blood Clearance of DT(GSHMe)_2_

**Fig 5:**
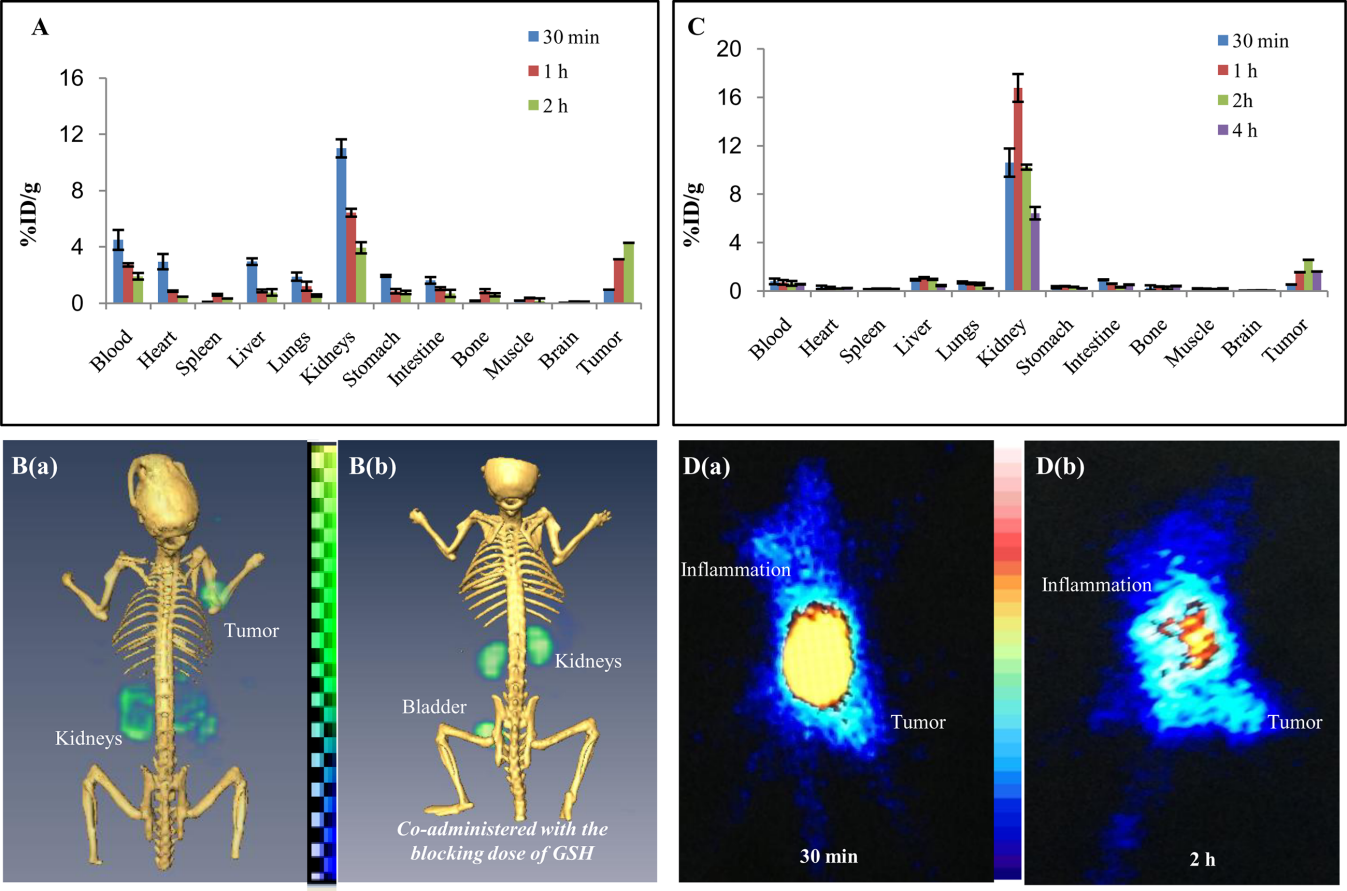
*In vivo* biodistribution and scintigraphy DT(GSHMe)_2_ on experimental models. *In vivo* biodistribution and scintigraphy of DT(GSHMe)_2_ on experimental models. A) Bar graph representing biodistribution of ^68^Ga-DT(GSHMe)_2_ on BALB/c mice bearing EA tumors. B) Coregistered microPET/CT whole body scan was performed on tumor models after 2 h of i.v. injection with and without co-administration of blocking dose of GSH. C) Biodistribution of ^99m^Tc-DT(GSHMe)_2_ in BALB/c mice bearing EA tumors. D (a & b) Whole body SPECT image of BALB/c mice bearing EA tumor in right hind limb and a site of inflammation in the left forelimb acquired at 30 min and 2 h post i. v. injection of ^99m^Tc-DT(GSHMe)_2_.

Biodistribution studies were performed for ^68^Ga-DT(GSHMe)_2_ in BALB/c mice bearing tumors. Distribution data revealed a similar trend to that compared with ^99m^Tc-DT(GSHMe)_2_ but in early time points cardiac activity was visible with ^68^Ga-DT(GSHMe)_2_ as the tracer. At 1 and 2 h, there was a decline in activity found in blood. (Fig 5A) Major accumulation of radioconjugate was observed in kidneys at 30 min with 11.077 ± 0.64% ID/g. Rapid washout of tracer was seen 1 h post injection. Liver shows uptake of the radiotracer 2.95 ± 0.236% ID/g and 0.868 ± 0.098% ID/g, as observed at 30 min and 1 h respectively. Uptake was insignificant which was well correlated with SPECT and PET images.*In vivo* biodistribution was also done for ^99m^Tc-DT(GSHMe)_2_ in tumor bearing BALB/c mice 30 min, 1, 2 and 4 h post i.v. injection of the radiotracer. Major accumulation was seen in kidneys with 16.78 ± 1.148% ID/g within 1 h. Furthermore, rapid washout of the radioactivity from kidneys was observed at 4 h with 6.43 ± 0.51% ID/g. At 1 and 2 h, minimal radioactivity 1.09 ± 0.059% and 0.96 ± 0.046% ID/g was found associated with liver tissue, which almost cleared off within 4 h. (Fig 5C) The comparison of the biodistribution with different isotopes was done using two tailed t- test and P <0.05 for all the organs except for the heart where significant difference was found in the accumulation of radioactivity where P>0.05.

**Fig 6:**
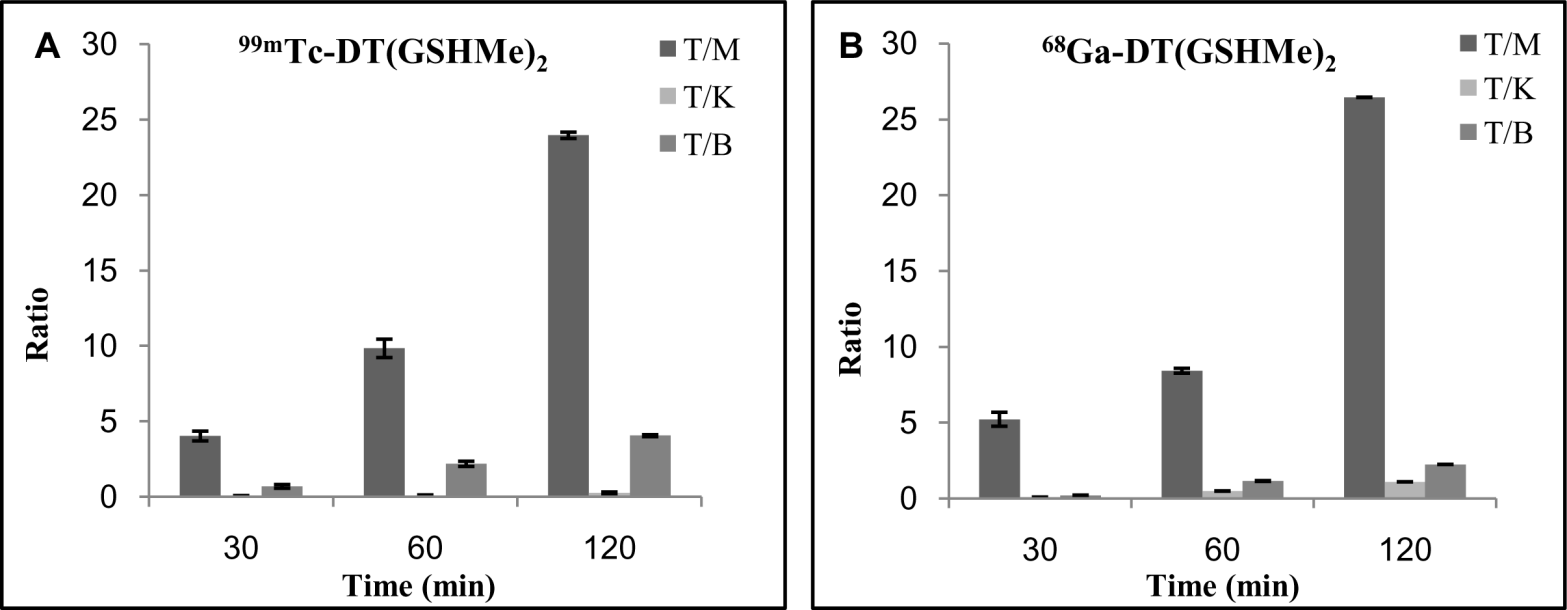
Tissue distribution ratios relative to tumor tissue. Ratio of the radioactivity distributed in different tissue to that of tumor at various time intervals for (A) ^99m^Tc-DT(GSHMe)_2_ and (B) ^68^Ga-DT(GSHMe)_2_ is represented graphically. All data were computed as mean ±SEM.

Fig 6A and 6B is a time dependent graph, depicts the ratios viz. tumor to muscle (T/M), tumor to blood (T/B), tumor to liver (T/L) and tumor to kidney (T/K) derived from the biodistribution data of ^99m^Tc-DT(GSHMe)_2_ and ^68^Ga-DT(GSHMe)_2_. A vivid retention of activity was observed in tumor tissue at 2 h and a high tumor/muscle ratio of 23.967 and 26.44 was achieved at 2 h for ^99m^Tc-DT(GSHMe)_2_ and ^68^Ga-DT(GSHMe)_2_ respectively. *In vivo* blood kinetics of the tracer was done to study the bioavailability of the radiolabeled tracer in the blood. Rapid blood clearance of the radiolabeled tracer from the circulation was observed with almost 14.85 ± 1.50% and 2.07 ± 0.269% activity retained in blood at 0.25 h and 4 h respectively. The biological half-life obtained was t_1/2_ (Fast): 8.8 min and t_1/2_ (Slow): 15.44 which inferred that 50% of the activity was eliminated in early 8.8 min. (See supplementary information for the graph).

### Scintigraphic studies

#### Micro PET/CT small animal imaging

Observations from small animal microPET experiments performed in athymic mice bearing BMG-1 tumors, post i.v. injection of ^68^Ga DT(GSHMe)_2_ are depicted in Fig 5B (a & b). All the images were acquired at 2 h post injection of the radiolabeled tracer. Significantly high uptake of ^68^Ga-DT(GSHMe)_2_ was evident on the tumor site. Data revealed that tracer eliminated from the body mainly by the renal route of excretion which was concomitant with the results obtained in biodistribution study. (Fig 5A) GGT target specificity of ^68^Ga-DT(GSHMe)_2_ was substantiated by blocking experiment. For blocking experiment, native GSH (reduced) was injected in excess dose of ~5 mg/Kg body weight for 1 h. 95.57% block was seen at tumor site confirming the target specificity. Semi quantitative volumetric ROI analyses were done using AMIDE 1.0.4 software. The analyses were suggestive of high target to non target ratio at the tumor site with minimal background activity. The tumor to contralateral muscle ratio was found to be 9.33, whereas tumor to kidney ratio was 0.93. (See supplementary information for ROI table).

#### SPECT imaging

Fig 5D (a & b) shows representative scintigraphic image of xenografted mouse model bearing EAT tumor in hind limb and an induced inflammation at fore limb, were obtained 30 min and 1 h post IV injection of ^99m^Tc-DT(GSHMe)_2_ 29.6 ± 1.8 MBq. SPECT data revealed accumulation of tracer both at inflammation and tumor site 30 min post i.v. injection. Significant differences were visualized in early and late images with substantial amount of radiotracer concentration at tumor site at 2 h. High kidney and bladder uptake depicted the renal route of excretion of the radioconjugate.

## Discussion

Understanding the biochemical processes and signaling pathways in cancer biology is the key requisite in pharmaceutical research to develop target specific radio tracer for the purpose of imaging or therapeutics. The concept of *in vivo* imaging is based on ligand-receptor interaction which lies in choosing an appropriate target which is found to be invariably expressing in the diseased condition. In this work we have designed DT(GSHMe)_2_ as the ligand for the target enzyme Gamma Glutamyl Transferase (GGT). GGT plays an essential role in providing growth advantage to proliferating cells [14].

There is evidence that GGT is up regulated in malignant cells and by producing reactive oxygen species causes tumor progression towards more aggressive phenotypes associated with a worse prognosis. This will aid in accurate interpretation of diagnostic capability of the PET and SPECT tracer targeting GGT expression. In this respect, radiolabeled DT(GSHMe)_2_ has been used for non-invasive molecular imaging, which is an interesting tool for early detection of rapidly growing tumors. Glutathione monoethyl ester has properties that are suitable for use in a cellular delivery system for GSH. Use of Glutathione monoethyl ester is preferred over methylated ester or other esters because, the intracellular cleavage products, like ethanol (in case of mono ethyl ester) is less likely to be toxic than methanol or higher alcohols [21].

Polyamino polycarboxylate ligand DTPA has two sites for conjugation of peptide glutathione monoethyl ester. DTPA offers stable radiochemistry with ^99m^Tc and is quickly cleared from the body via the renal routes. Defined radiochemistry is indispensable for the target receptor—ligand interaction. The synthesis of DT(GSHMe)_2_ was a two step synthesis, where in the first step glutathione is esterified at the carboxyl group of glycine. In the reaction the equivalents of reactant are optimally controlled so as to avoid the formation of glutathione diethyl ester. This is followed by conjugating Glutathione mono ethyl ester to DTPA, again in stochiometrically controlled reaction to avoid monomer formation. The appreciably good reaction yield of 82.5% is obtained for the final product.

*In vitro* evaluation of drug on cell lines reveals DT(GSHMe)_2_ to be non toxic and correlated well in terms of ligand integrity and specificity towards GGT. Immuno-histochemical analysis of tumor excised from mice bearing xenografted EAT and BMG-1 tumors, have shown positive expression of GGT in sections. BMG-1 tumors show a high positive expression of GGT while EA tumors show a moderately positive expression of GGT. High expression of GGT in tumor in comparison to contralateral muscle is also evident from the western blotting studies.

The ligand offers stable radiochemistry with ^99m^Tc and ^68^Ga as shown by high radiolabeling efficiency and radiochemical purity, under optimized condition and was found to be stable in vitro in saline upto 24 h. Radiocomplexation of DT(GSHMe)_2_ with ^99m^Tc and ^68^Ga is cost effective and facile since the isotopes are eluted from the generator. DT(GSHMe)_2_ was effectively transported inside the cell and was validated by the time course experiments giving the maximum rate of transport Vmax of 0.478 μM/min. From cell binding and kinetics analysis, it was found that Km>>Kd showing directional uptake of DT(GSHMe)_2_ inside the cell. Blood kinetics and biodistribution of the drug when radiolabeled with ^99m^Tc and ^68^Ga clearly indicated its fast clearance from the body via the renal route which is indispensably required for any radio tracer for high contrast. First order kinetics was observed for ^99m^Tc-DT(GSHMe)_2_ from the blood kinetics experiments.

Biodistribution studies were performed to study the accumulation pattern of the tracer in the body. The data revealed the accumulation of drug in blood and heart in early 15 and 30 min. At late intervals most of the activity cleared from the body. Retention of activity followed a declining trend in the kidneys for the later time points. The purpose of the work was to image tumor via PET/SPECT modalities, so the drug was used at pharmacologically ineffective concentration which was sufficient enough to image the tumor. To test the hypothesis, PET/SPECT imaging was performed on mice bearing xenografted tumors. The high tumor uptake with a rapid washout from the body except for the target site, led to excellent visualization of tumor at 2 h post injection. In Inflammation-tumor model, accumulation of activity at both inflammation and tumor site was seen 30 min post injection, which is attributed to the retention of activity in the blood pool. Later, at 2 h tracer retention was only seen at tumor site suggestive of tumor specificity.

DT(GSHMe)_2_ could not be tagged to heavy metals(α or β-emitters) owing to its possibility of demetallation in biological system. But it is worthwhile to note that preclinical investigations revealed the putative efficacy of DTGSMe as a diagnostic tool for tumor imaging which could be further explored for its clinical applications. For effective clinical application purpose, we intend to translate the work for human use after ethical clearance. A single vial lyophilized, endotoxin free kit containing 500 μg of DT(GSHMe)_2_, 200 μg of stannous chloride at pH 7.0 was tested on animal models in house. The kit could be reconstituted with sterile ^99m^Tc-pertechnate (185MBq-740MBq) with high radiolabeling yield of the order of >98%. Validation of the kit for human use is further warranted.

To conclude, GGT is an important marker in many tumor types and can be exploited to develop imaging and therapeutic agents. DT(GSHMe)_2_ is possibly transported via GGT system and is a good candidate for imaging malignant lesions and prognostication. Preclinical investigation of the homodimeric tripeptidic system has shown its potential as an imaging probe in terms of its stable and defined radiochemistry with ^99m^Tc and ^68^Ga. In preclinical models, DT(GSHMe)_**2**_ shows high and specific accumulation at tumor site versus inflammation site. Therefore it could prove to be a better system for PET/SPECT imaging and prognostication of malignant tumor. DT(GSHMe)_2_ could be promising candidate for translational molecular imaging plausibly with high utility in clinics. This could be further extended to therapy by tagging it to ^99m^Tc/ ^188^Rhenium matched pairs as theranostic agent.

## Supporting Information

S1 Fig Mass spectrum of Glutathione monoethyl ester (GSHMe). Desired peak of 335.12 was found at [M+H] 336.3.
(TIF)

S2 Fig Mass spectrum of Diethylene triaminpentaacetic acid bis glutathione monoethylester [DT(GSHMe)_2_]. Desired peak of C_38_H_61_N_9_O_20_S_2_: 1027.35 was found at [M+H] 1028.5.
(TIF)

S3 Fig Analytical HPLC chromatogram of DT(GSHMe)_2_. Peak of DT(GSHMe)_2_ was observed with retention time of 5.5 min with 66.16% purity.
(TIF)

S4 Fig Radio TLC of DT(GSHMe)_2_. The two peaks above show radiolabeled ^68^Ga-DT(GSHMe)_2_ and free ^68^Ga-citrate. Above is the chromatogram of labeled compound without purification. Below is the chromatogram of Ga-68 eluted from the generator.
(TIF)

S5 Fig Radio HPLC of ^68^Ga-DT(GSHMe)_2_. Concurrent peak in HPLC UV detector and HPLC gamma detector at Retention time 3.5 min confirmed the labeling with ^68^Ga.
(TIF)

S6 Fig Saturation binding curve ^99m^Tc- DT(GSHMe)_2_ on EAT cells. (TIF)

S7 Fig Blood Kinetics of ^99m^Tc- DT(GSHMe)_2_. Kinetics results reveal t_1/2_ fast of 8.8 min and t_1/2_ slow of 15.44 h.
(TIF)

S1 Table Semi quantitative ROI analysis. Region of interest of same size ellipsoid was drawn for three tissue types and were determined by AMIDE image analysis software.
(TIF)
