# Supplementary figures and images for: Preclinical Evaluation of a Potential GSH Ester Based PET/SPECT Imaging Probe DT(GSHMe)2 to Detect Gamma Glutamyl Transferase Over Expressing Tumors
Source: PLoS One. 2015 Jul 29;10(7):e0134281. doi: 10.1371/journal.pone.0134281 (PMC4519333; doi:10.1371/journal.pone.0134281)

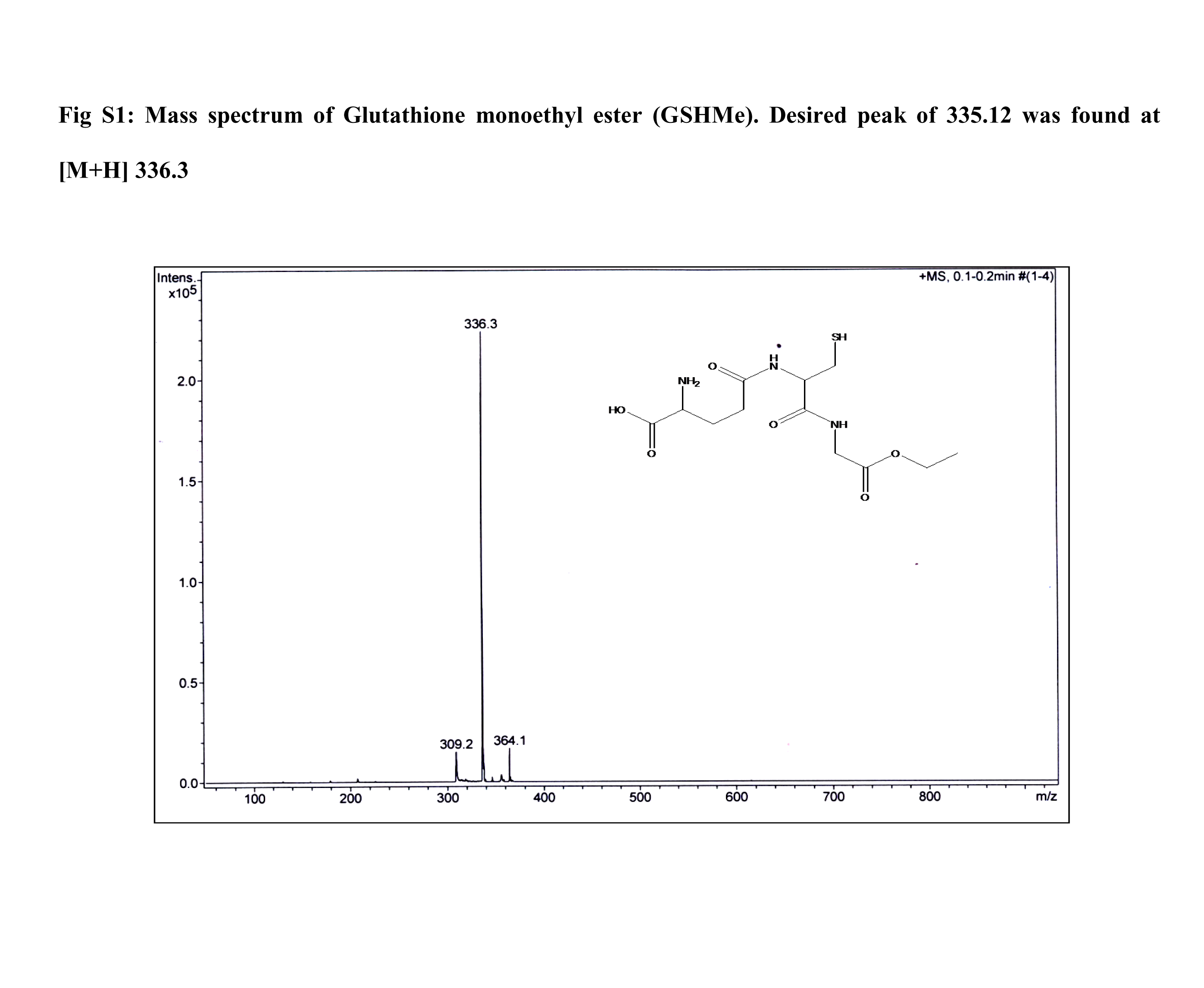

Supplement: S1 Fig — Desired peak of 335.12 was found at [M+H] 336.3. (TIF) [file pone.0134281.s001.tif]

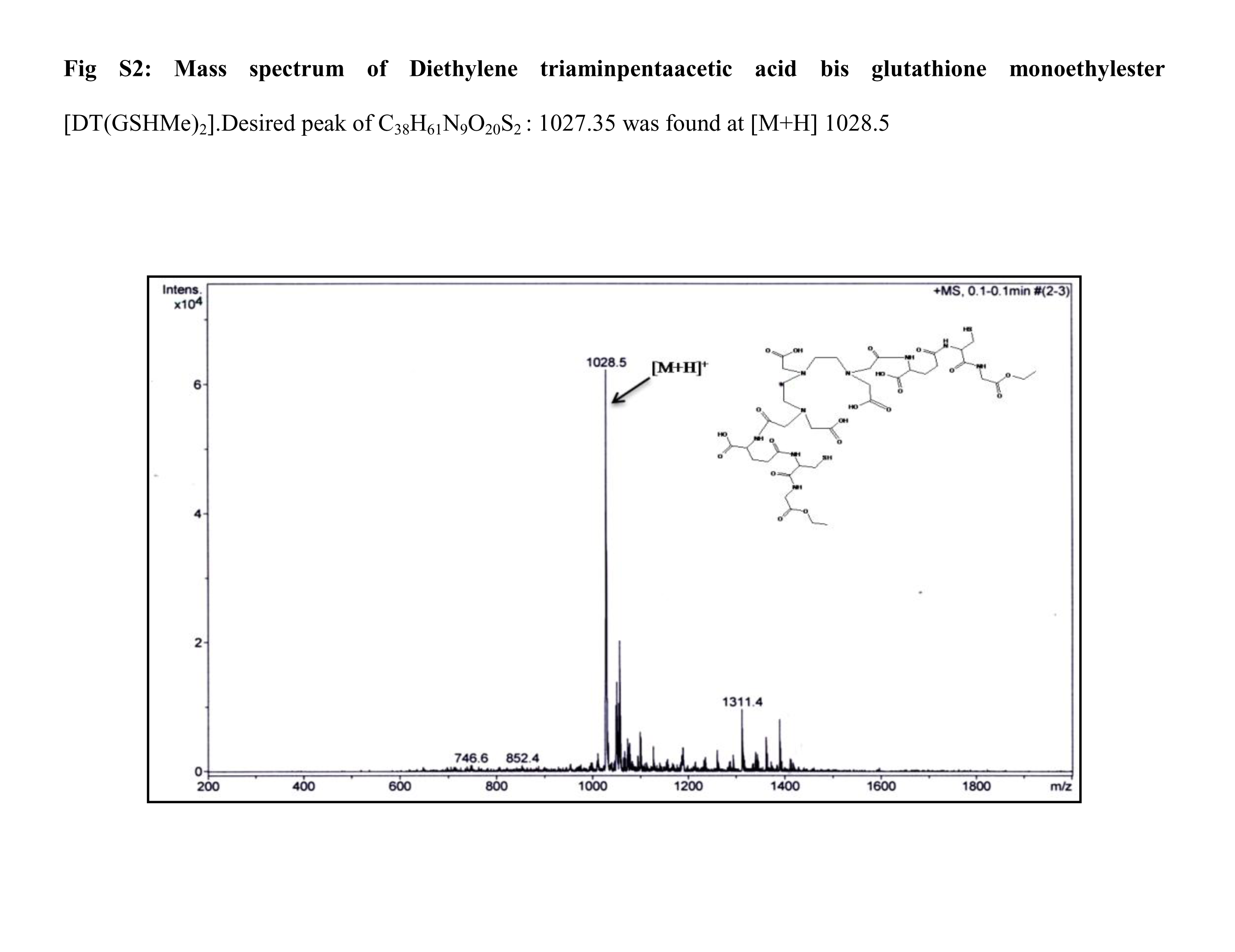

Supplement: S2 Fig — Desired peak of C38H61N9O20S2: 1027.35 was found at [M+H] 1028.5. (TIF) [file pone.0134281.s002.tif]

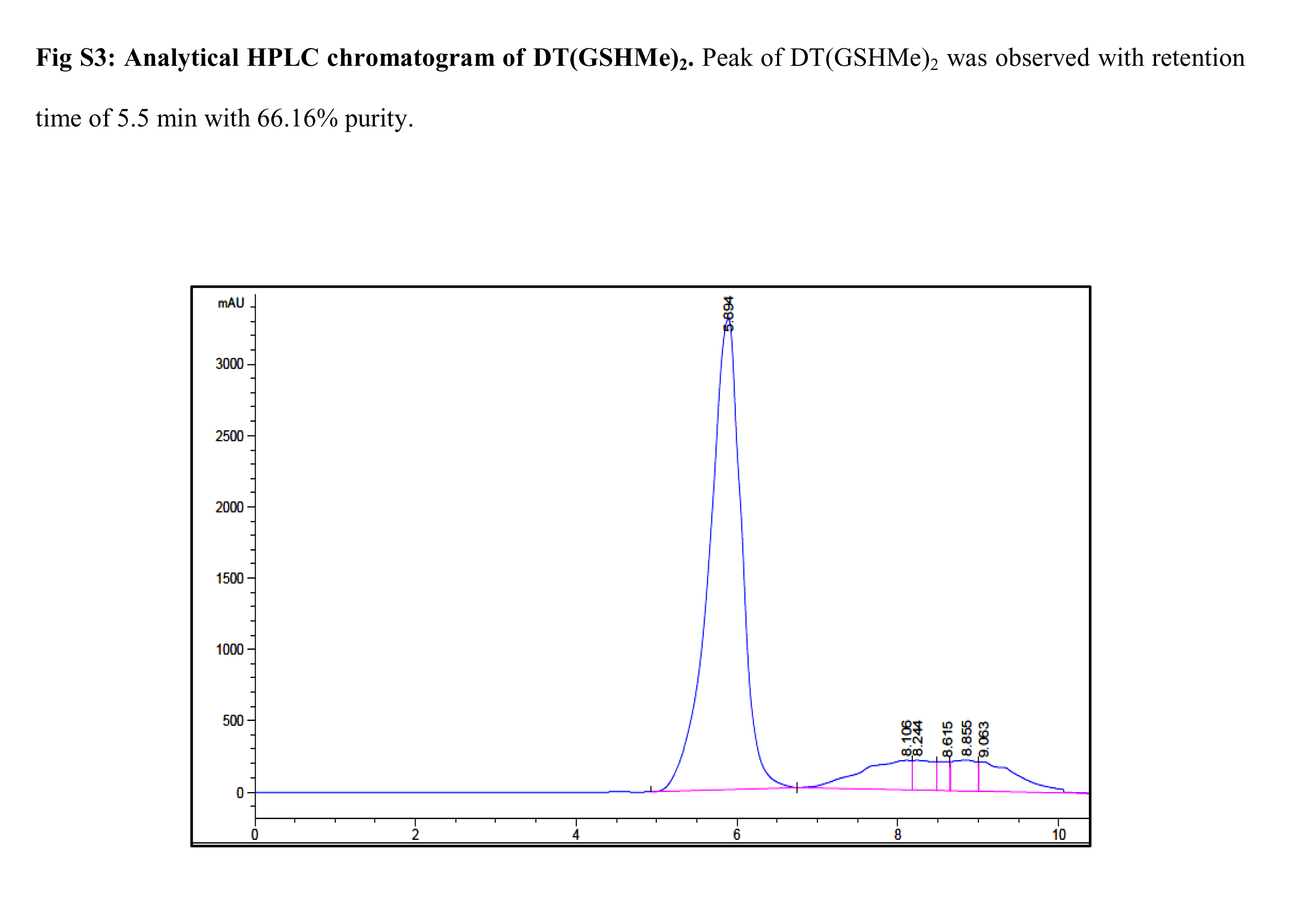

Supplement: S3 Fig — Peak of DT(GSHMe)2 was observed with retention time of 5.5 min with 66.16% purity. (TIF) [file pone.0134281.s003.tif]

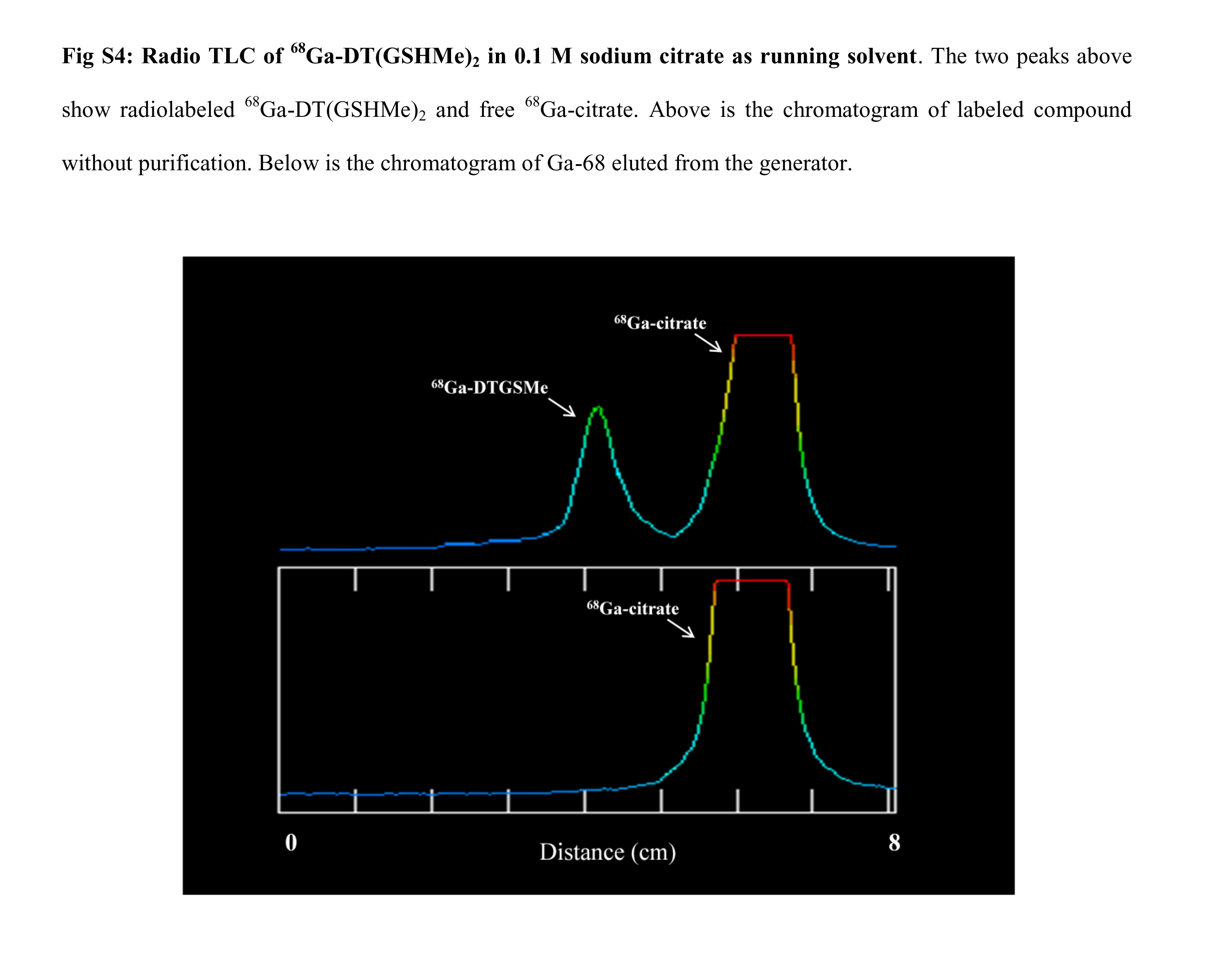

Supplement: S4 Fig — The two peaks above show radiolabeled 68Ga-DT(GSHMe)2 and free 68Ga-citrate. Above is the chromatogram of labeled compound without purification. Below is the chromatogram of Ga-68 eluted from the generator. (TIF) [file pone.0134281.s004.tif]

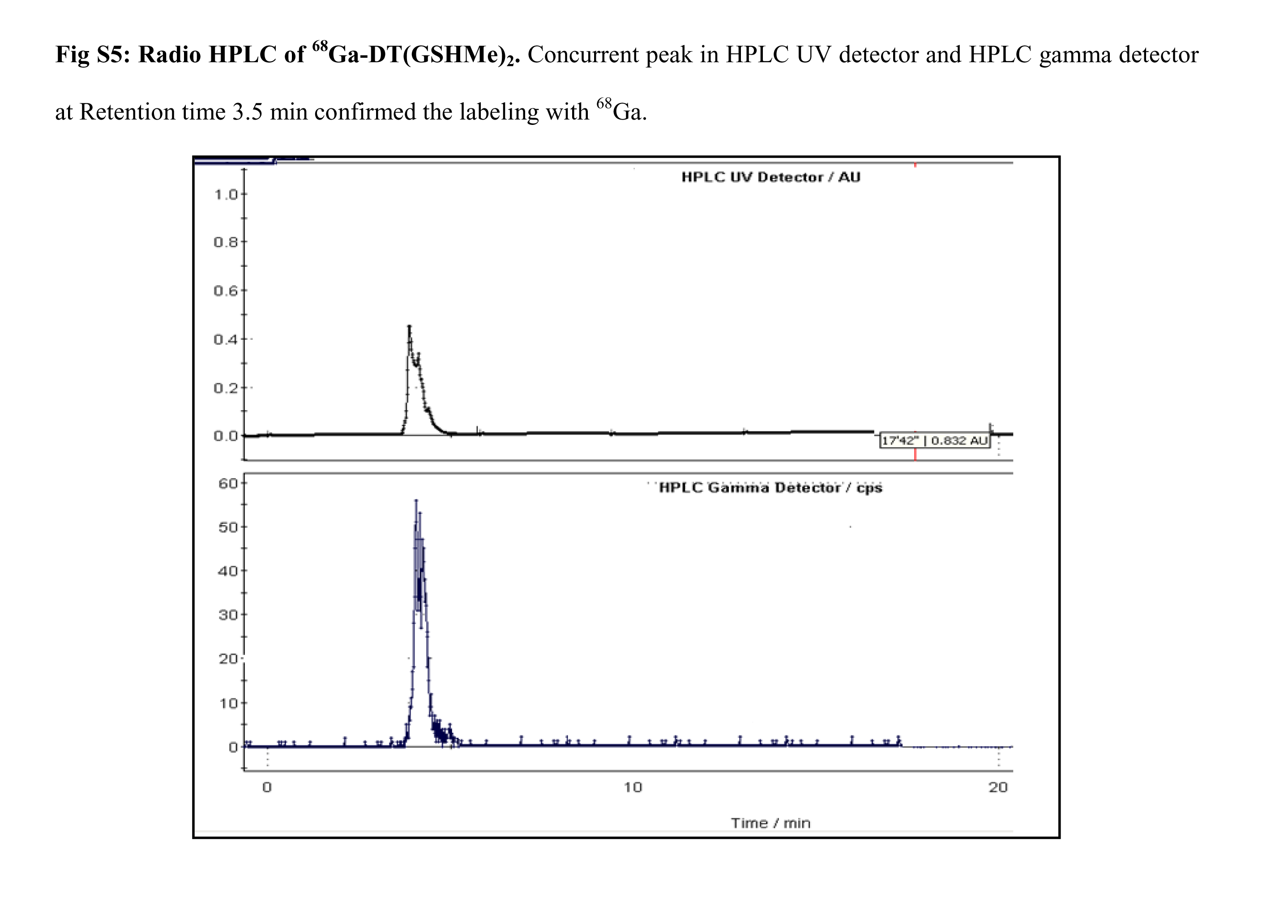

Supplement: S5 Fig — Concurrent peak in HPLC UV detector and HPLC gamma detector at Retention time 3.5 min confirmed the labeling with 68Ga. (TIF) [file pone.0134281.s005.tif]

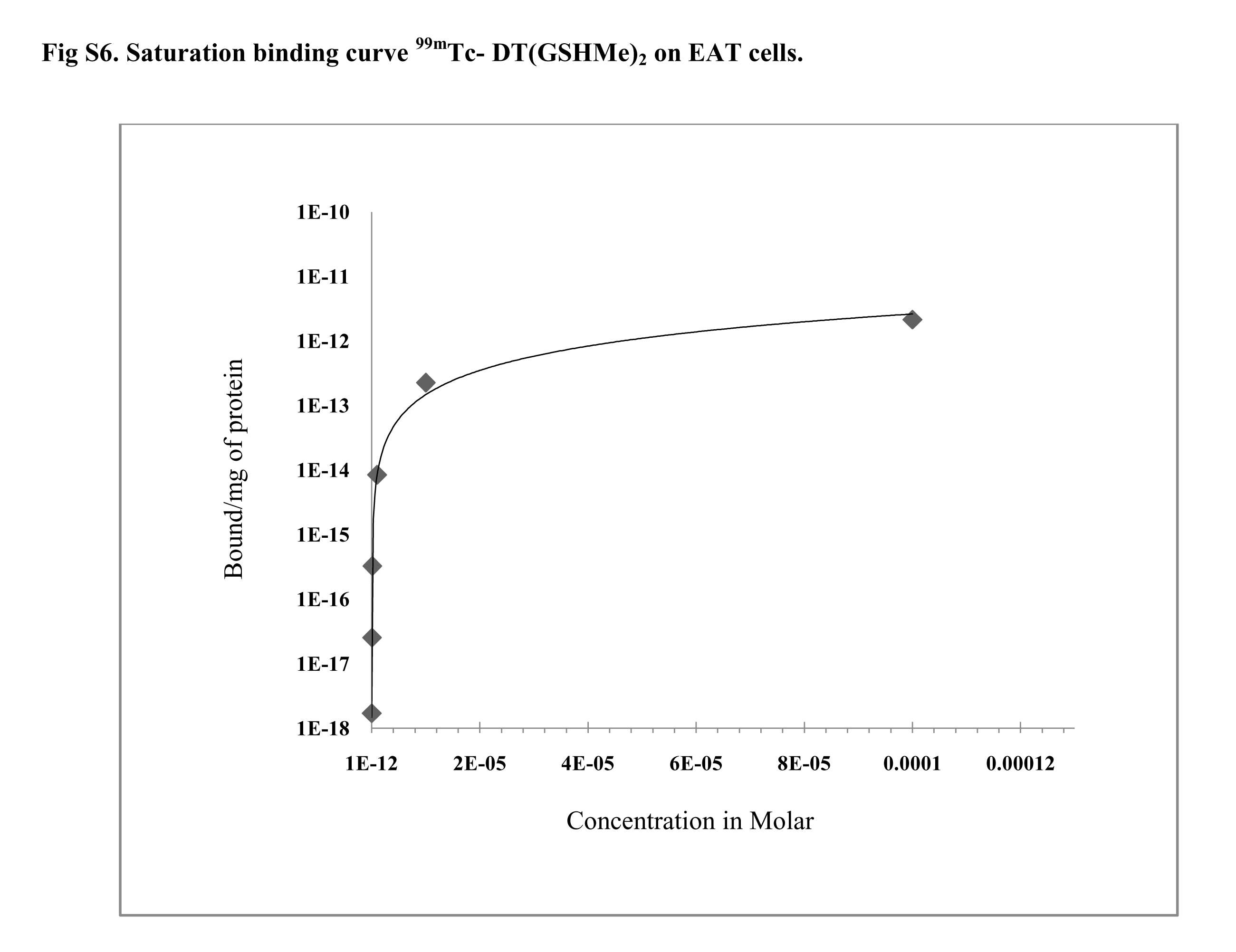

Supplement: S6 Fig — (TIF) [file pone.0134281.s006.tif]

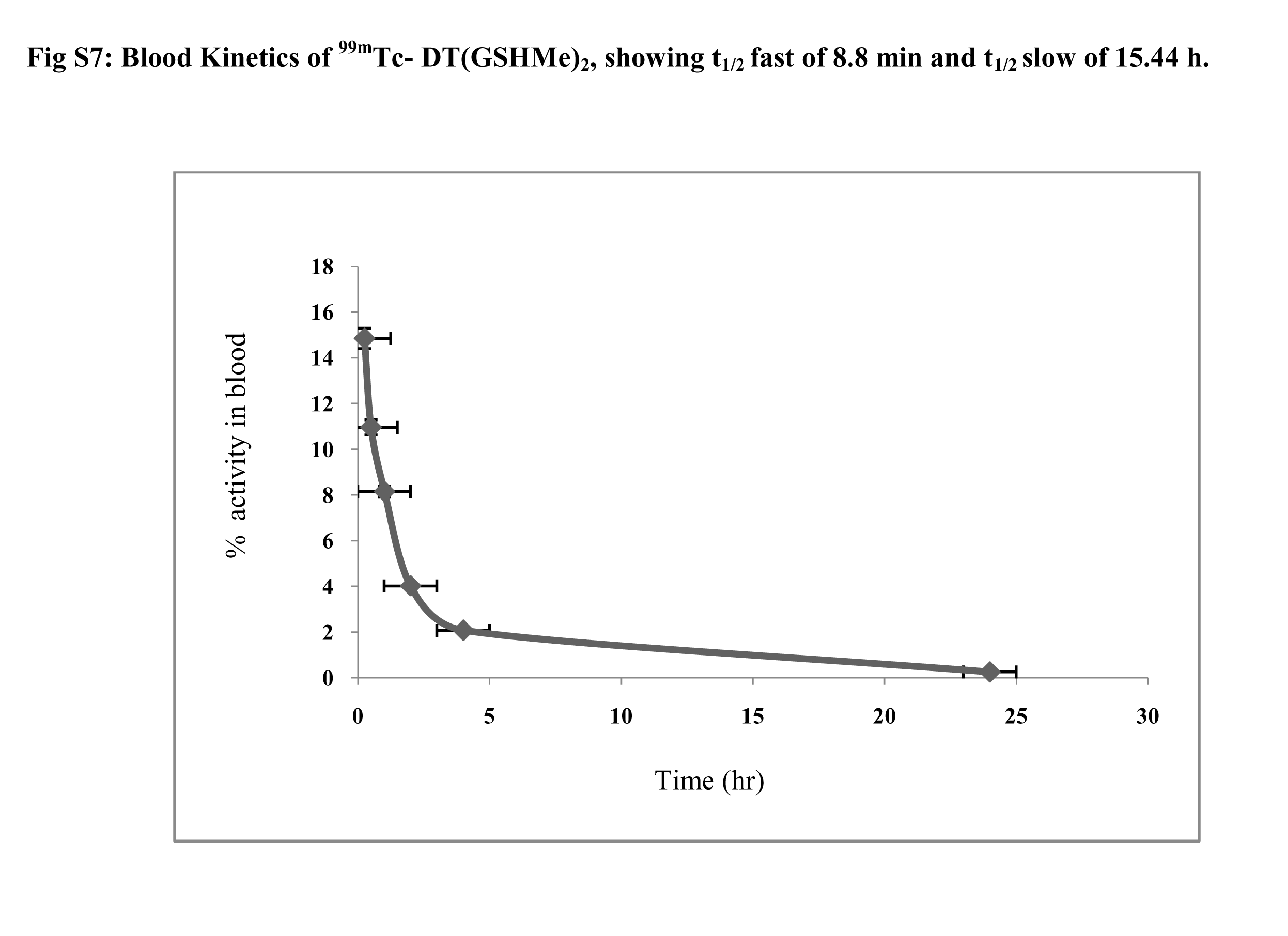

Supplement: S7 Fig — Kinetics results reveal t1/2 fast of 8.8 min and t1/2 slow of 15.44 h. (TIF) [file pone.0134281.s007.tif]

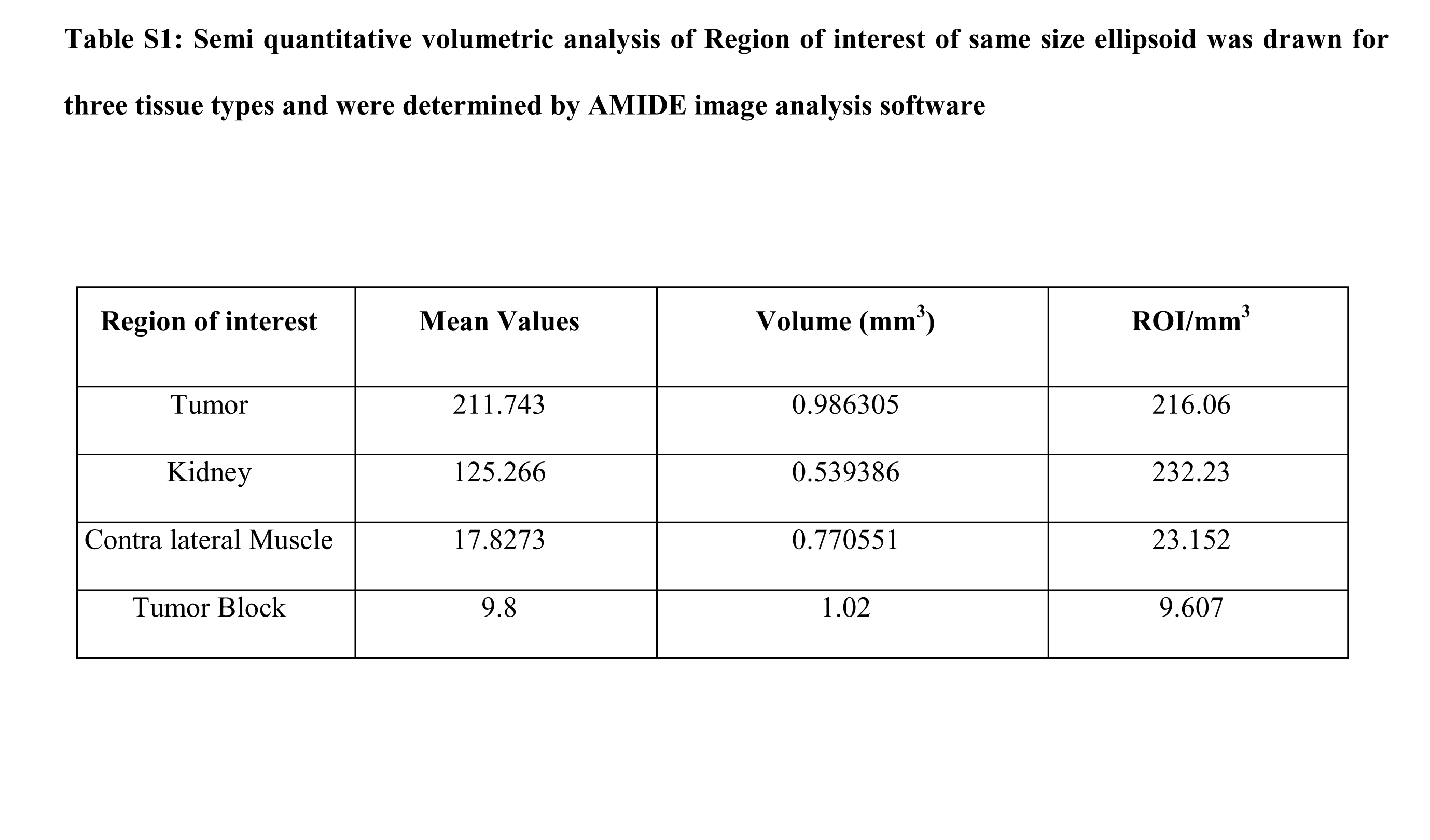

Supplement: S1 Table — Region of interest of same size ellipsoid was drawn for three tissue types and were determined by AMIDE image analysis software. (TIF) [file pone.0134281.s008.tif]
